# Effectiveness of Polidocanol in the Treatment of Venous Malformations: A Meta-Analysis

**DOI:** 10.3389/fped.2022.925318

**Published:** 2022-07-28

**Authors:** Wei Hu, Zhuang Liu, Jiali Sun, Liang Wang, Dan Song, Lei Guo

**Affiliations:** ^1^Children’s Hospital Affiliated to Shandong University, Jinan, China; ^2^Shandong Provincial Clinical Research Center for Children’s Health and Disease, Jinan, China; ^3^Department of Vascular Anomalies and Interventional Radiology, Jinan Children’s Hospital, Jinan, China

**Keywords:** sclerotherapy, efficacy, vascular malformations, VMS, polidocanol

## Abstract

**Objective:**

The aim of this study was to investigate the efficacy of polidocanol against venous malformations (VMs).

**Methods:**

Studies reporting the treatment of VMs using polidocanol (published until February 15, 2020) were reviewed in the Embase and PubMed databases. After excluding the same literature, part of the studies were excluded by reading the title, abstract, full text. Eleven studies (with 287 participants) that fulfilled the inclusion criteria were included. Systematic meta-analysis was performed using Reviews Manager 5.2, and a fixed-effects model was used to calculate the pooled effective rate of polidocanol against VMs and the 95% confidence intervals (CI).

**Results:**

Lesion reduction of more than 50% was considered effective. A total of 287 patients were treated, and treatment in 271 was considered effective. The efficacy of polidocanol was 0.89 (95% CI = 0.83–0.93). Heterogeneity among the studies was small (*I*^2^ = 0%, *P* = 0.47). T The funnel plot was roughly symmetric.

**Conclusion:**

Our study suggested that polidocanol is effective in the treatment of VMs. VMs at different sites can be treated without serious complications. Therefore, we have reason to believe that polidocanol is a safe and an effective drug for VMs.

## Highlights

- Venous malformations (VMs), the most common vascular malformations, are vascular anomalies that mostly occur in the skin and the mucosa. Surgical intervention, which is associated with a high recurrence rate, has gradually been replaced by percutaneous sclerotherapy due to its superior efficacy and lower invasiveness. Several sclerosants have been reported for the treatment of VMs. Polidocanol is one such sclerosant with a mechanism of action that involves killing of the vascular endothelial cells.
- Though previous studies have reported the use of polidocanol in the treatment of VMs in various sites, these are mostly single-center experiences. Therefore, we have reviewed the existing literature from various centers for assessing and comprehensively presenting the safety, efficacy, and stability of polidocanol in the treatment of VMs.

- Our study suggested that polidocanol has an efficacy of 96% in the treatment of VMs. Its usage is not associated with any serious complications. Polidocanol appears to be safe and effective in the management of VMs.

## Introduction

Venous malformations (VMs) are slow-flowing, non-proliferative vascular anomalies that mostly occur in the skin and the mucosa (1). These constitute the most common types of vascular malformations. Although most cases of VMs are asymptomatic, some cases are symptomatic (2). VMs have physical and psychological impacts on the patients: Their physical impact is characterized by pain, swelling, and dysfunction, while their psychological impact is characterized by changes in the appearance arising from these malformations. Surgical resection was previously considered as an important conventional treatment for VMs (3); however, due to the complex structure of the lesion, which often infiltrates the surrounding tissues, a recurrence of VMs was often observed even after their curative resection (4). Therefore, over the past 2 decades, owing to its superior efficacy and lesser invasiveness, percutaneous sclerotherapy has gradually replaced surgical resection as the main treatment for VMs (5). A variety of sclerosing agents have been reported for the treatment of VMs; common agents include foam sclerosants, polidocanol, sodium tetradecyl sulfate, anhydrous ethanol, and bleomycin (6). An ideal sclerosant should have a high efficacy, manageable complications, and wider application. Although each sclerosant has a different mechanism of action, all cause a shrinkage of the VM’s nidus and resolve its symptoms (5). Polidocanol is an effective sclerosing agent, and its main mechanism of action involves killing the vascular endothelial cells (7). Previous studies have reported the use of polidocanol in the treatment of VMs in various sites; however, these were mostly single-center studies. Therefore, in this study, we have reviewed the existing literature for assessing the safety, efficacy, and stability of polidocanol in the treatment of VMs.

## Materials and Methods

### Literature Review and Search Strategy

Using a computer-based retrieval system, 2 researchers explored the PubMed and the Embase databases for literature on polidocanol usage available from the databases’ dates of launch to February 15, 2020. Subject terms and free text were used for data retrieval. The languages were restricted to English and Chinese. The following search terms were used: “[polidocanol (supplementary concept) OR polidocanol] AND [‘vascular malformations’ (mh) OR VMs].”

### Inclusion Criteria

**FIGURE 1:**
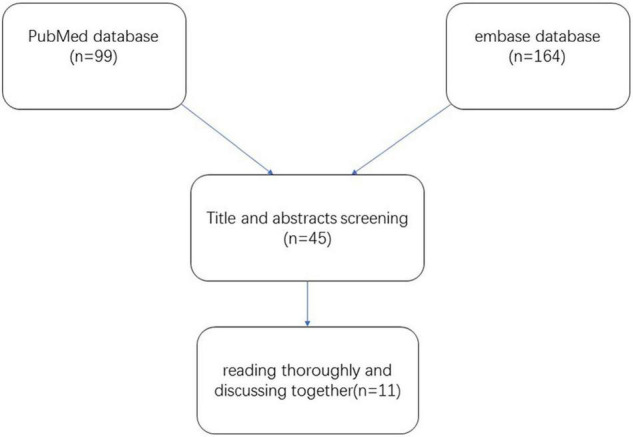
Flowchart of literature search and study selection.

We included publications on the use of polidocanol in the treatment of VMs. Only published studies were taken into consideration. All publications included adults and/or children. These publications detailed studies on the efficacy of polidocanol against VMs; the extent of lesion reduction was used as a criterion for efficacy. A detailed flowchart demonstrating the study selection process is shown in Figure 1.

### Study Selection and Data Extraction

Two researchers independently screened the search results, extracted data using pre-established criteria, and assessed the risk of bias. Disputes arising during data processing were resolved by 2 persons through consultation. Studies were selected based on the title and the abstract; the full text was referred to later for further screening. The following information was extracted from each study: (1) the name of the first author, (2) year of publication, (3) focal location, (4) number of cases with effective treatment, (5) sample size of the study, (6) types of studies, (7) duration of follow-up, (8) the concentration of polidocanol administered, (9) Clinical Evaluation, (10) Mean No. of sessions, (11)Mean number of treatments, (12) Number of Complication, (13) Number of recurrent cases. The study quality was evaluated with reference to the methodological index for non-randomized studies (MINORS) Collaboration tool (8). The same was used for evaluating the risk of bias.

### Data Synthesis and Analysis

Meta-analysis was performed using the Reviews Manager 5.2 software. For dichotomous outcomes arising from the absence of a control group, 95% confidence intervals (CIs) were estimated. A fixed-effects model was used for the present meta-analysis. Heterogeneity among the studies was evaluated using *I*^2^ statistics; *I*^2^-values greater than 50% indicated high heterogeneity. Furthermore, the funnel plot asymmetry was determined to assess publication bias. Probability values of < 0.05 were considered as statistically significant.

This study was a control with no dichotomous data, and the outcome measure was the effective rate of treatment, data on the type of ratio should be calculated by (9). Assuming an effective rate of P, a sample size of N, and an effective number of X, when the assumption of normal distributed data were not met, it is calculated as:


P=ln(x/(n-x))

$$\mathrm{SE}\left(p\right)=\sqrt{1/x+1/\left(n-x\right)}$$

The Revman software was used to calculate that the results obtained needed a transformation calculation to obtain the pooled effective rate and 95% CI, and the conversion formula was as follows:

Conversion of effect measures:


Pf=OR/(1+OR)

95% CI lower limit transformed:


$$LL=LL_{OR}/\left(1+LL_{OR}\right)$$

95% C_*I*_ upper limit transformed:


$$UL=UL_{OR}/\left(1+UL_{OR}\right)$$

## Results

### Literature Search, Description, and Quality of the Included Studies

A total of 263 potentially relevant studies were identified in the literature search. Around 99 publications were excluded by reviewing the titles and abstracts. Around 119 publications were further excluded upon reviewing the full articles. After thoroughly reading and discussing the remaining 45 articles, 34 publications were excluded. Finally, 11 publications with 305 patients were selected for the meta-analysis (10–20). The quality of the included publications was relatively low.

### Characteristics of the Included Studies

All included publications detailed single-center studies and were published between 2000 and 2020. The sample size ranged from 7 to 70 subjects. The basic characteristics of the studies are summarized in Table 1. Around 3 publications focused on VMs of the head and the neck, while 1 publication reported retrobulbar VMs. The remaining publications covered parts of the body such as the limbs, genitalia, and trunk. Of the 11 publications reviewed, 2 were prospective and 9 were retrospective in nature, as shown in Table 1.

**TABLE 1:** Baseline characteristics of studies included in the meta-analysis.

References	Location	Number of cases with effective treatment	Sample size of the study	Type	Follow-up	Drug concentration	Clinical evaluation	Mean no. of sessions	No. of complication	Complication rate	No. of recurrent
Chen et al. (18)	Head and Neck	70	70	Retrospective study	1, 3, and 6 months after the final treatment	1%	Size reduction	2.14	Swelling × 114, pain × 2, Epidermal extravasations × 1	78%	0
Yang et al. (11)	Retrobulbar VMs	6	7	Prospective study	3 months after treatment	1%	Size reduction, The mean exophthalmos score; The mean intraocular pressure; The mean VAS score	1.29	Swelling, transient blurred vision × 3, temporary pain × 1	44%	0
Ali et al. (13)	Head, Neck, Trunk, Upper/Lower extremity	34	37	Prospective study	6 months after the last session	1%	Size reduction, Improvement of clinical symptoms (pain, bleeding, dysfunction, appearance)	3.5	No major complications; Temporary pain × 34, Phlebitic reaction × 29, Cutaneous necrosis × 2	50%	3
Gulsen et al. (17)	Llimb	16	19	Retrospective study	2 months after the last session of treatment	2%	Size reduction, improvement of clinical symptoms	2.4	/	/	0
Chen et al. (14)	Head and Neck	10	11	Retrospective study	1, 3, and 6 months after the final treatment	1%	Size reduction	3.9	Swelling × 11, snoring × 1, pain with swallowing × 1	30%	1
Yamaki et al. (10)	Genitalia, Head, NeckUp, per/Lower extremity, Others	23	28	Retrospective study	1 month	3%	Size reduction	1	Temporary pain × 23, Swelling × 21, Epidermal necrosis × 3, Hemoglobinuria × 4	82%	5
Kumar et al. (20)	Head, Neck, Trunk, Upper/Lower extremity	52	53	Retrospective study	6 months	3%	Size reduction, Pain improvement, Patient satisfaction	/	Temporary pain × 23, Swelling × 46	/	0
Kumar et al. (16)	Masseter	17	17	Retrospective study	6–26 months, mean 15.9 months	3%	Size reduction	2.05	Swelling × 17, Vomit × 1	52%	0
Mukul et al. (15)	Tongue	14	15	Retrospective study	3–4 weeks after treatment, 5 years	3%	Size reduction	/	Swelling, Partial tongue mucosa necrosis	/	0
Jain et al. (19)	Limb	9	9	Prospective study	6 months	1%	Size reduction	3.67	Superficial erythema and induration of the skin × 2	6%	0
Niu et al. (12)	Oropharynx	20	21	Retrospective study	2–29 months	3%	Size reduction	2.5	Swelling × 13, Fever × 3, Local ulcer × 2	34%	0

### Efficacy Rate

**FIGURE 2:**
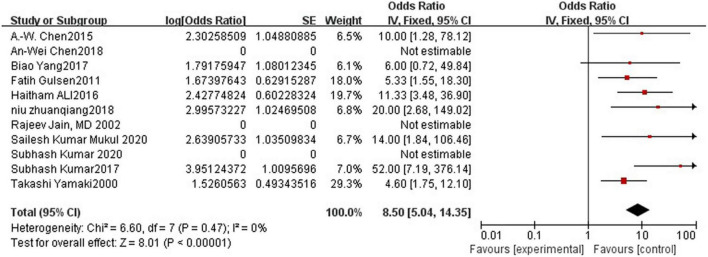
Forest plot for polidocanol effectiveness.

Polidocanol effectiveness was 86%. It was reported in all included studies and was measured by a reduction in the VM lesion size. Low heterogeneity was observed between all statistical analyses (*I*^2^ < 50%), as shown in Figure 2.

Complication rate was 47%.

### Adverse Events

**FIGURE 3:**
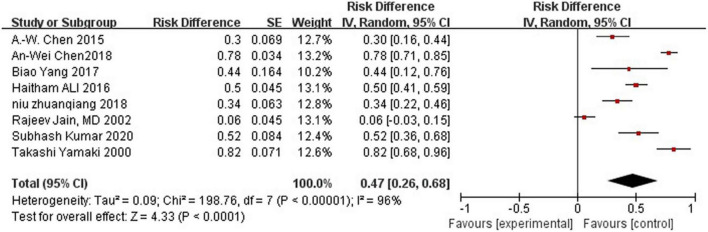
Forest plot for complications.

Most studies reported postoperative complications. Eight studies reported swelling, pain, and skin necrosis, which disappeared within a week following surgical intervention. One study reported a small amount of postoperative pigmentation that disappeared within a short time, while another reported postoperative proteinuria. However, one study did not report any obvious complications. Because some literatures did not provide detailed data of complications, they were excluded when calculating the incidence of complications. High heterogeneity was observed between all statistical analyses (*I*^2^ > 50%), the random effect model is used, as shown in Figure 3.

### Publication Bias

**FIGURE 4:**
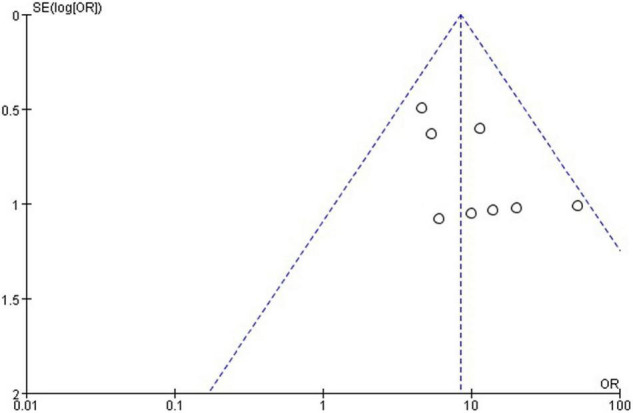
Funnel plot of polidocanol effectiveness.

**FIGURE 5:**
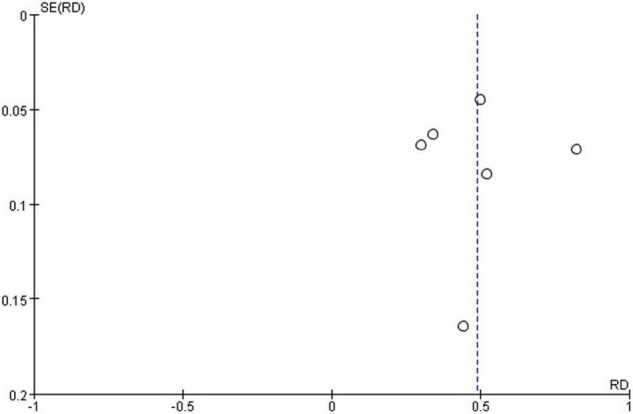
Funnel plot of complications.

The funnel plot generated from the studies reporting polidocanol efficacy was roughly symmetrical (Figure 4). This may be attributed to the smaller sample sizes, which produced a smaller effect. The funnel plot generated of complication rate was roughly symmetrical (Figure 5).

## Discussion and Conclusion

VMs are congenital malformations of the vascular system (21). Their symptoms are known to persist from childhood to adulthood. The vast majority of VMs do not heal spontaneously and their symptoms worsen gradually.

Surgery, once the mainstream treatment for VMs, has gradually been replaced by sclerotherapy due to its limitations involving functional and aesthetic sequelae and high recurrence rates (3). However, some scholars believe that surgery cannot be completely ruled out, because sclerotherapy alone cannot completely eliminate residual fibrosis and phleboliths (4).

Compared to surgical treatment, percutaneous sclerotherapy is associated with a relatively lower trauma and has a lower cost (5). Polidocanol, a sclerosant, is widely used in the treatment of VMs. It was initially developed in France in the 1950s as a local anesthetic that could be used as a liquid or foam. In March 2020, it was approved by the US Food and Drug Administration as a sclerosing agent.

Our literature survey revealed that the VMs of the trunk, limbs, head, and neck in children and adults were treated with polidocanol with an efficacy of 86%. The definition of “effective” varied across literature. Therefore, in this study, an effective rate was determined on the basis of the effect indicators (change in lesion volume after treatment) in the literature studied. Symptoms were also observed to be important effect indicators; almost all publications (whether included in this study or not) reported that the symptoms of most patients alleviated or disappeared after sclerotherapy. Improvement of clinical symptoms after treatment was also mentioned in the literatures included in this study.

Only a few meta-analyses are available on the treatment of VMs with polidocanol. Five studies have focused on the use of polidocanol in the treatment of venous diseases including gastric variceal bleeding, telangiectasias of the lower limbs, and esophageal varices. Only one meta-analysis reported the efficacy of polidocanol for the treatment of VMs (22). The publications included in this study were limited to case studies in China and included another sclerosing agent, Lauromacrogol, which is similar to polidocanol and is mostly practical in China. The significance of our analysis lies in the fact that it reviewed literature on the use of polidocanol in the treatment of VMs at various sites.

Some issues require further investigation. Firstly, the specific details of the patient receiving sclerotherapy such as the concentration of polidocanol administered, the time between consecutive treatments, and the type and location of the lesion should be considered in the analysis. In the literatures included in this study, different concentrations of polidocanol were used to treat VMs, which did not explain the reason, possibly due to the local drug supply. In addition, the time interval between two treatments reported in different literatures is also different, which may be due to the doctor’s judgment of the patient’s condition. All the literatures were not sufficiently detailed to report the complications of polidocanol, which may be due to the relatively minor and rapid recovery of the related complications. Different literatures refer to different criteria to evaluate complications, however, most of the literatures included in this study only described the symptoms of complications and did not grade them. We counted all cases of complications (23). The degree of complications was evaluated, and most complications were grade 1, one study explicitly mentioned that patients’ complications reached grade 2 (11). Secondly, the studies included in this meta-analysis reported different concentrations of polidocanol for treating VMs. The reason behind this remains unclear; It may be due to the local drug supply. Thirdly, the time interval between two treatments differed across the studies; this may be due to the doctor’s judgment of the patient’s condition. Thirdly, efficacy was not assessed in a uniform fashion. In the literature included in this review, VMs occurred in many parts of the body, such as the head and neck, trunk, extremities, oropharynx, etc., imaging is difficult to estimate lesion volume in VMs because of irregular morphology, and evaluation of treatment efficacy is judged by whether lesions disappear or become less symptomatic in most of this literature, some literatures use the change of volume or lesion diameter to evaluate the treatment effect. This inconsistency of evaluation criteria may affect the overall evaluation of efficacy. Furthermore, the studies included were insufficiently detailed regarding the complications of polidocanol; this may be due to the relatively minor and rapid recovery of the associated complications. Due to the large individual differences in patients with VMs, the optimal dose of polidocanol remains unknown. To address these issues, more well-designed randomized controlled trials are needed. Based on the current research, polidocanol is an effective and a safe sclerosant for treating VMs; no publication has concluded otherwise.

This meta-analysis provides strong evidence that supports the use of polidocanol in the treatment of VMs.

## Data Availability Statement

The original contributions presented in this study are included in the article/supplementary material, further inquiries can be directed to the corresponding author.

## Author Contributions

LG conceived and designed the meta-analysis. ZL and WH searched literatures and extracted data. JS was responsible for writing and revising articles. LW and DS analyzed the data. All authors reviewed the manuscript and approved the submitted version.

## Conflict of Interest

The authors declare that the research was conducted in the absence of any commercial or financial relationships that could be construed as a potential conflict of interest.

## Publisher’s Note

All claims expressed in this article are solely those of the authors and do not necessarily represent those of their affiliated organizations, or those of the publisher, the editors and the reviewers. Any product that may be evaluated in this article, or claim that may be made by its manufacturer, is not guaranteed or endorsed by the publisher.
