# Corrigendum: Effectiveness of polidocanol in the treatment of venous malformations: A meta-analysis

**DOI:** 10.3389/fped.2022.1011511

**Published:** 2022-09-21

**Authors:** Wei Hu, Zhuang Liu, Jiali Sun, Liang Wang, Dan Song, Lei Guo

**Affiliations:** ^1^Children's Hospital Affiliated to Shandong University, Jinan, China; ^2^Shandong Provincial Clinical Research Center for Children's Health and Disease, Jinan, China; ^3^Department of Vascular Anomalies and Interventional Radiology, Jinan Children's Hospital, Jinan, China

**Keywords:** sclerotherapy, efficacy, vascular malformations, VMS, polidocanol

In the published article, there was an error in the author list, and authors “Zhuang Liu” and “Wei Hu” were listed in the incorrect order. The corrected author list appears below.

“Wei Hu, Zhuang Liu, Jiali Sun, Liang Wang, Dan Song and Lei Guo”

The authors apologize for this error and state that this does not change the scientific conclusions of the article in any way. The original article has been updated.

## Publisher's note

All claims expressed in this article are solely those of the authors and do not necessarily represent those of their affiliated organizations, or those of the publisher, the editors and the reviewers. Any product that may be evaluated in this article, or claim that may be made by its manufacturer, is not guaranteed or endorsed by the publisher.

